# Distinct neural signaling characteristics between fibromyalgia and provoked vestibulodynia revealed by means of functional magnetic resonance imaging in the brainstem and spinal cord

**DOI:** 10.3389/fpain.2023.1171160

**Published:** 2023-05-22

**Authors:** Gabriela Ioachim, Howard J. M. Warren, Jocelyn M. Powers, Roland Staud, Caroline F. Pukall, Patrick W. Stroman

**Affiliations:** ^1^ Center for Neuroscience Studies, Queen’s University, Kingston, ON, Canada; ^2^ Department of Medicine, University of Florida, Gainseville, FL, United States; ^3^ Department of Psychology, Queen’s University, Kingston, ON, Canada; ^4^ Department of Biomedical and Molecular Sciences, Queen’s University, Kingston, ON, Canada; ^5^ Department of Physics, Queen's University, Kingston, ON, Canada

**Keywords:** fMRI, brainstem, spinal cord, pain, human, chronic, fibromyalgia, provoked vestibulodynia

## Abstract

**Introduction:**

Fibromyalgia and provoked vestibulodynia are two chronic pain conditions that disproportionately affect women. The mechanisms underlying the pain in these conditions are still poorly understood, but there is speculation that both may be linked to altered central sensitization and autonomic regulation. Neuroimaging studies of these conditions focusing on the brainstem and spinal cord to explore changes in pain regulation and autonomic regulation are emerging, but none to date have directly compared pain and autonomic regulation in these conditions. This study compares groups of women with fibromyalgia and provoked vestibulodynia to healthy controls using a threat/safety paradigm with a predictable noxious heat stimulus.

**Methods:**

Functional magnetic resonance imaging data were acquired at 3 tesla in the cervical spinal cord and brainstem with previously established methods. Imaging data were analyzed with structural equation modeling and ANCOVA methods during: a period of noxious stimulation, and a period before the stimulation when participants were expecting the upcoming pain.

**Results:**

The results demonstrate several similarities and differences between brainstem/spinal cord connectivity related to autonomic and pain regulatory networks across the three groups in both time periods.

**Discussion:**

Based on the regions and connections involved in the differences, the altered pain processing in fibromyalgia appears to be related to changes in how autonomic and pain regulation networks are integrated, whereas altered pain processing in provoked vestibulodynia is linked in part to changes in arousal or salience networks as well as changes in affective components of pain regulation.

## 1. Introduction

Chronic pain affects up to 18% of women and can have significant negative impacts on a person's quality of life and relationships (1). Fibromyalgia (FM) and provoked vestibulodynia (PVD) are two chronic pain conditions which affect 2% and 7 to 12% of the population, respectively. Yet, despite their impact, the underlying mechanisms are still poorly understood. FM presents as widespread, diffuse musculoskeletal pain, and symptoms include both heightened pain sensitivity (hyperalgesia) and painful responses to innocuous tactile stimuli (allodynia) (2–4). In contrast, PVD is the most common subtype of vulvodynia, and is described as a provoked pain resulting from pressure to the vaginal entrance (5–7). Although the primary pain is localized to the vestibular area, women with PVD have exhibited hypersensitivity to stimuli in other areas of the body as well (8, 9). Given that both FM and PVD pain have behavioral findings indicative of altered central sensitization (9–21) and autonomic dysfunction (22–26), some studies have described a degree of comorbidity of FM and PVD (27–30). One study by Ghizzani et al. demonstrated that women with FM may be more sensitive in the vulvar area regardless of whether they also experience provoked pain (27). Phillips et al. showed that women with PVD who also fulfill the criteria for FM experience more severe pain than women with PVD alone (28), and Pukall et al. demonstrated that women with PVD have an increased number of tender points compared to healthy control participants (29). These important commonalities between FM and PVD may indicate common underlying mechanisms.

Despite these links, few neuroimaging studies have compared these conditions. Recent functional magnetic resonance imaging (fMRI) studies in the brainstem and spinal cord have explored how pain processing in FM (12, 17) and PVD (21) is altered compared to healthy controls. While one fMRI study in the brain has examined both PVD and FM participants (15), it was limited by including FM participants as a control group. These investigations provided important information about pain processing in PVD, but they did not report the same depth of information about FM pain. Directly comparing these two conditions could expand our understanding of the underlying pain mechanisms.

In the present fMRI study, we investigated FM and PVD participants and healthy controls with data from prior studies. This dataset al.lowed us to examine brainstem and spinal cord connectivity during painful stimulation as well as when participants were anticipating the stimulus. We have previously demonstrated that pain modulation includes important continuous neural activity that is engaged before, during, and after a painful stimulus (31–33). We hypothesized that there would be specific differences in how pain modulation networks are integrated with other brainstem regions such as those involved in autonomic regulation networks in FM compared to PVD. We also hypothesized that there would be similar altered pain processing elements in FM and PVD groups compared to healthy controls.

## 2. Methods

The current study incorporated data from two large-scale fMRI studies on fibromyalgia (24) and provoked vestibulodynia respectively (21). Both studies received approval from the Queen's University Research Ethics Board. Only the details relevant to the current study from the brainstem/spinal cord acquisition sessions are discussed and analyzed here.

### 2.1. Fibromyalgia data

In a prior fMRI study, 15 women with fibromyalgia (mean age 46 ± 13 years) who fulfilled the 1990 and 2016 FM criteria (34) were recruited from online and community advertisements, as well as 15 healthy women who did not experience chronic pain (mean age 39 ± 10 years). All participants were free of major illnesses, neurological disorders, and contraindications for MR imaging such as metallic implants, and were not taking any centrally acting medications. Other medications were permitted provided the dosage had been stable for at least 3 months prior to the study. This was done to avoid any potential withdrawal effects of medications during the study. Because conventional pain medications do not significantly alleviate fibromyalgia pain (35), this is not expected to have a strong influence on the study findings.

Participants completed a set of demographic questionnaires and assessments. Included in this were the State-Trait Anxiety Inventory (STAI) (36), and Beck Depression Inventory (BDI) (37), as FM is known to be comorbid with anxiety and depression in many cases (3). We also included the Pain Catastrophizing Scale (PCS) (38) to assess whether individual reports of pain ratings were associated with tendency to catastrophize painful sensations or ruminate on past pain experiences. The Short-Form McGill Pain Questionnaire-2 (SF-MPQ-2) (39) was used to assess the intensity and descriptors participants used for their pain.

### 2.2. Provoked vestibulodynia data

Participants with and without PVD were recruited from the general community and from contact databases of the Sexual Health Research Laboratory (Queen's University) as well as advertisements to health care providers who may be treating women with vulvodynia (e.g., gynecologists, pelvic health specialists). To qualify for the study, women with PVD had to report idiopathic provoked pain to the vaginal entrance during activities involving contact with the vaginal entrance, which was confirmed via a gynecological (40). This is necessary because PVD is a diagnosis of exclusion (6), therefore care must be taken to rule out other potential causes of pain such as infections. For the brainstem/spinal cord study, 16 women with PVD were recruited (mean age 30 ± 10 years), and 16 pain-free women (mean age 30 ± 10 years) were age-matched to the PVD participants within 5 years. Healthy controls were also matched to the PVD participants in regard to hormonal contraceptive use (yes/no). All participants were free of any contraindications for MR imaging, did not have any major neurological disorders, and were not taking any centrally acting medications.

All participants completed demographic questionnaires as well as questionnaires about their pain experiences, and anxiety and depressive symptoms. Similar to the fibromyalgia data described above, the questionnaires included assessments of state and trait anxiety [STAI (36),], depression [BDI (37)], pain catastrophizing [PCS (38),], and pain descriptors [SF-MPQ-2 (39),].

### 2.3. Quantitative sensory testing and “sham” MRI

**Figure 1:**
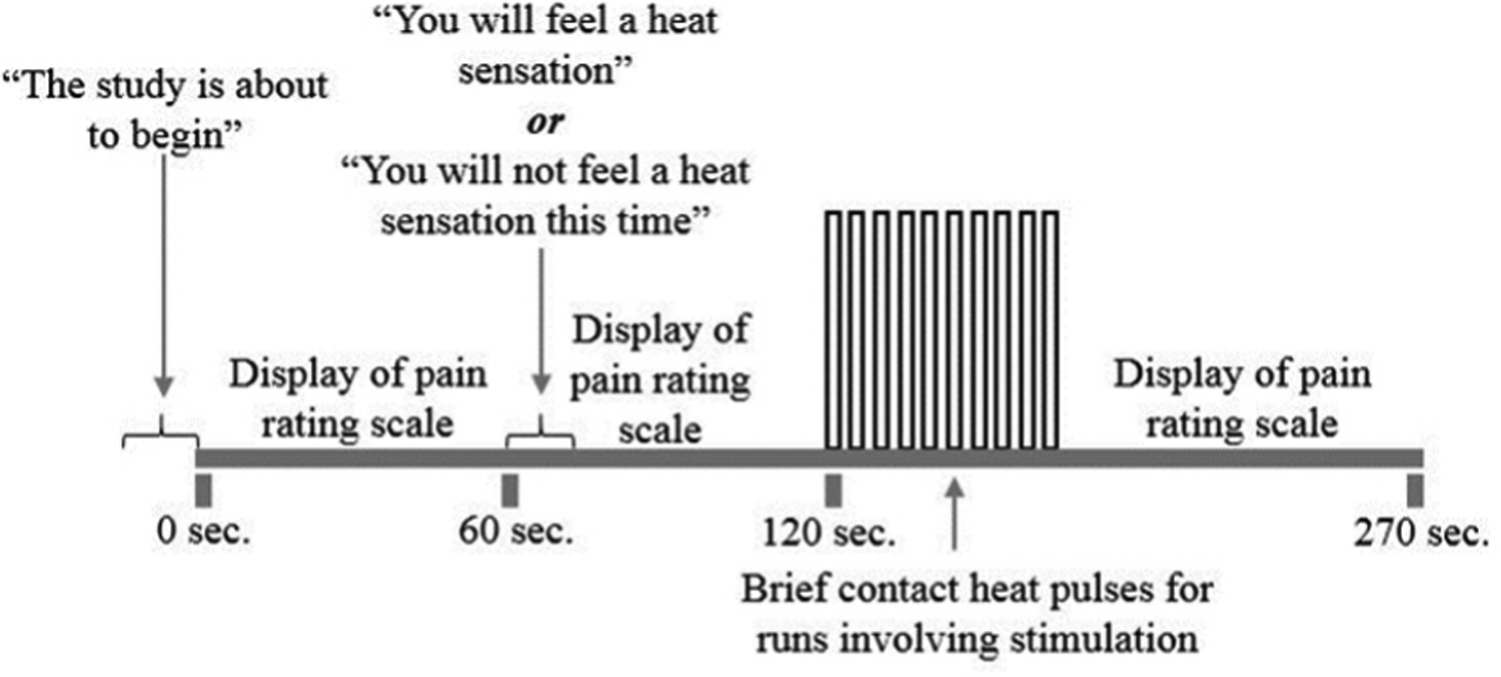
Task paradigm for the fibromyalgia and provoked vestibulodynia data. Periods of interest that were analyzed in the current study include “Expecting Pain” (centered at 90 s, or 1 min 30 s) and “Experiencing Pain” (centered at 135 s, or 2 min 15 s). While the larger data set also included runs without the painful stimulus, only the runs where the noxious stimulus was applied were analyzed in the current study.

The fibromyalgia and provoked vestibulodynia data were collected by the same research teams as part of a larger inter-lab collaboration. Therefore, all participants received the same stimulus and protocol training, quantitative sensory testing (QST) and imaging sessions. First, participants were familiarized with the predicable noxious heat stimulus paradigm of the study, termed the “threat and safety” paradigm. A visual breakdown of this paradigm is given in Figure 1.

The participants were trained to rate their pain on a 100-point numerical pain intensity scale (NPS) (41) with descriptive labels at 10 point increments (0 = no sensation, 10 = warm, 20 = a barely painful sensation, 30 = very weak pain, 40 = weak pain, 50 = moderate pain, 60 = slightly strong pain, 70 = strong pain, 80 = very strong pain, 90 = nearly intolerable pain, 100 = intolerable pain). They were told they would not be subjected to any stimuli that could harm them, and that they were free to stop the stimulation any time during the study if they felt the need to. The noxious heat stimulus was delivered by a custom-made MRI-compatible robotic contact-heat thermal stimulator (RTS-2). This device consisted of a 3 cm square aluminum thermode in a plexiglass housing. The participants rest their right hand on the housing, and the thermode is pneumatically advanced from the housing to make contact with the skin of their hand on the thenar eminence and is then retracted into the housing. The temperature and timing of the thermal contacts were precisely controlled by the researcher with a custom-written MATLAB software. A heat stimulus was chosen in order to remain consistent with recent pain research in the spinal cord (12, 31, 42–44) and repeated contacts are used because they produce a dynamic stimulus that includes temporal summation of pain (i.e., “wind-up”) which can be used to probe central sensitization (17, 18, 45) and has been shown in previous studies to produce a robust response in fMRI data (12, 17, 21, 33, 43, 44, 46–48). While higher sensitivity to heat pain compared to controls has been observed in FM (3) but not PVD (49), both conditions have been speculated to involve changes in central pain processing and central sensitization and may respond differently to this paradigm compared to pain-free control participants (12, 15, 18, 20).

All participants underwent a 1-hour session which included practicing rating their pain on the NPS and familiarizing themselves with the stimulus and the paradigm timing. Each participant received a series of stimuli intensities in the same order (46, 50, 44, and 48 °C), each consisting of 10 heat contacts delivered over 30 s. In between each trial participants were given two minutes of recovery time to allow the temperature of the skin to return to normal and any aftersensations to dissipate before another stimulus was applied. During these trials, participants were asked to rate their pain for each contact out loud. Following these trials, the temperature of the stimulus was adjusted for each participant to produce a pain rating of 50 on the NPS, although temperatures never exceeded 52 °C to avoid any potential tissue damage. Participants were blinded to this calibration and were never told which stimulus temperature they were receiving. The session then concluded with a practice run in a sham MRI where participants practiced the full 4 min and 30 s paradigm for the imaging session. The sham MRI provides an environment similar to the MRI and allows the participants to experience the imaging environment. In the sham run, they were asked to rate each contact on the NPS mentally, to avoid excessive movement due to speaking during imaging, and to remember the first and last rating for the run. After the run, participants were asked to report their first and last pain ratings.

The stimulation paradigm (Figure 1) spanned 4 min and 30 s and consisted of 1 min of baseline during which time participants did not know whether a stimulus would be applied to their hand. At 1 min they were informed via a rear-projection screen whether or not the run would include the painful stimulus, followed by 1 min when participants knew what to expect. At the 2-minute mark, the noxious heat stimulus was delivered (during the no-pain runs no stimulus was applied). Stimulation consisted of 10 heat contacts delivered over a span of 30 s. Participants spent the remaining 2 min after stimulation waiting for the trial to end before providing their pain intensity ratings to the first and last contacts.

### 2.4. Functional MRI data acquisition

#### 2.4.1. fMRI paradigm

The imaging protocol was identical for all participants, regardless of the study group. Each imaging session consisted of 10 fMRI acquisitions (i.e., “runs”) of 4.5 min each (Figure 1), separated into 5 “Pain” runs in which participants felt the noxious heat stimulus, and 5 “No Pain” runs in which no stimulus was applied. In each run, participants were informed at 1 min mark via a rear-projection screen whether or not that particular run would include the heat stimulus. They could therefore anticipate the timing of the stimulus or relax for the remainder of the time with no stimulus during the No Pain runs. After each run that included a stimulation period, participants were asked via a 2-way intercom to report their pain ratings for the first and last contacts using the NPS which was displayed to them throughout each run. While the “Pain” and “No Pain” runs were randomly interleaved for each participant, only the runs including the painful stimulus were analyzed for this current study. More information on the separate FM and PVD studies as a whole is published elsewhere (21, 24). During each Pain run, participants experienced 1 min of baseline, were informed that the run would contain a painful stimulus, experienced the 1-minute anticipation period, were delivered the 10 heat contacts over a period of 30 s, and then experienced another 2 min of baseline where they waited for the run to end (Figure 1). After each run, participants were asked via intercom to report their pain ratings for the first and last contact using the NPS which was displayed for them throughout the run.

#### 2.4.2. fMRI data acquisition

The functional MRI scans were performed on a Siemens 3 tesla MR system (Siemens Magnetom, Erlangern, Germany), which underwent an upgrade from a Siemens Magnetom Trio to a Siemens Magnetom Prisma Fit towards the end of data collection. All PVD data were collected on the Trio system, while the FM data were partially collected on the Prisma system as well. All study protocols, procedures, and personnel were consistent pre- and post-upgrade, and checks were performed with the FM data as well as additional volunteer data to ensure the quality of the data and the signal-to-noise ratio in the data sets were equivalent. No significant differences were found and all acquisition parameters were able to be maintained pre- and post-upgrade, and the FM data were pooled and analyzed together.

Participants were positioned with padding under the head, knees, and arms to provide a comfortable position and reduce muscle tension and motion. They could view the study instructions and NPS via a mirror on a rear-projection screen and could communicate with the researchers through an intercom between each run. Posterior head, neck, and spine receiver coils were used to detect the signal. Functional MRI data were acquired from the brainstem and spinal cord with a half-Fourier single-shot fast spin-echo sequence (HASTE) with BOLD contrast (50). The peripheral pulse was also recorded with an optical sensor on one finger of their left hand and was used to model physiological noise components. Localizer images were acquired in three planes to aid in slice positioning, and functional MR image data were acquired in 9 contiguous sagittal slices spanning from below the first thoracic vertebra to above the corpus callosum, with a 28 × 21 cm field-of-view and 1.5 × 1.5 × 2 mm^3^ resolution. This method has been shown to provide optimal image quality and BOLD sensitivity in the brainstem and spinal cord (43) and is consistent with our previous studies involving predictable noxious stimuli (12, 31, 33, 48). Imaging parameters included an echo time (TE) of 76 msec and a repetition time (TR) of 6.75 s/volume for optimal T_2_-weighted BOLD sensitivity and for consistency with our previous studies (12, 42, 51–53). Each 4.5-minute run included 40 volumes, and 5 runs including a noxious heat stimulus were acquired for each participant, resulting in 200 volumes of data per individual.

### 2.5. Data preprocessing and analysis

#### 2.5.1. Preprocessing

Spinal cord and brainstem fMRI data were preprocessed and analyzed using custom-written software (43), “spinalfmri9” (https://www.queensu.ca/academia/stromanlab/home/fmri-analysis-software) in MATLAB (MathWorks, Natick, MA, USA). Image data were converted from DICOM to NIfTI format and co-registered to correct for bulk body motion using the non-rigid 3D registration tool in the MIRT (Medical Image Registration Toolbox) package (54, 55). Images were then resized to 1 mm^3^ voxels and spatially normalized to a pre-defined anatomical template based on 356 participants, as described previously (42, 48). Physiological noise estimates were obtained from the recording of the peripheral pulse (which was synchronized to each fMRI time series), global noise models were estimated from predefined regions of white matter, and motion parameters obtained from the co-registration procedure were used as estimates for bulk movement. These noise models were then fitted to the data using a general linear model (GLM), and subtracted from the data to remove physiological noise (53).

#### 2.5.2. Analysis

For the current study we analyzed two time period (epochs) spanning 45 s each. One epoch was centered at the 1 min 30 s mark and is the period of time when participants knew to expect a painful stimulus but had not felt the stimulus yet (“Expecting Pain” period), while the other block was centered at 2 min 15 s, marking the period of time in which participants were experiencing the painful stimulus (“Experiencing Pain”). To reduce the number of comparisons and remain consistent with our previous studies, fMRI time-series data were extracted from 10 regions of interest (ROIs) which were defined using a probabilistic anatomical region map (12, 42, 56) which had been compiled from several atlases and published papers (57–61). The spinal cord region included the right dorsal quadrant of the 6th cervical spinal cord segment (C6RD), which was chosen because the noxious stimulus was applied to the thenar eminence of the right hand, corresponding with the C6 dermatome. Brainstem regions included the thalamus (Thal), hypothalamus (Hyp), periaqueductal gray matter (PAG), parabrachial nucleus (PBN), locus coeruleus (LC), nucleus tractus solitarius (NTS), nucleus raphe magnus (NRM), nucleus gigantocellularis (NGc), and dorsal reticular nucleus of the medulla (DRt). As these regions are not expected to carry out only one function at a time (31, 32, 62–64), they were each divided into 5 sub-regions using k-means clustering. Dividing our data into regions and sub-regions in this way reduces the number of comparisons for analyses and provides greater spatial precision by defining the clusters of voxels in each sub-region based on their functional characteristics. Clusters were defined using all of the data from all participants, and the same cluster definitions were then used to extract the data for each participant separately, for the subsequent analyses.

Both the FM and PVD studies included a group of healthy women as controls. Each group was age-matched to their respective chronic pain group, which resulted in a different average age for the two control groups. We performed both behavioral and connectivity analyses and found no major differences in questionnaire scores or connectivity networks between the two control groups in either the Expecting Pain or Experiencing Pain time periods. Therefore, the two control groups were combined into one overall healthy control group (HC) to simplify analyses and interpretation of results. This combination of groups allowed us to account for individual differences between participants while comparing HC to chronic pain groups.

#### 2.5.3. Structural equation modeling

**Figure 2:**
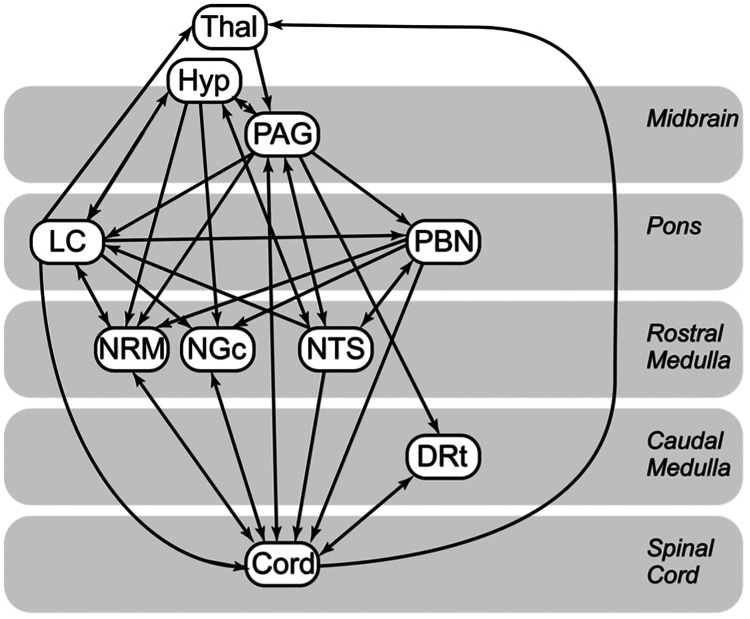
Anatomical model of the regions and connections used for the structural equation modelling (SEM) analysis. Arrow points indicate directions modeled for each connection.

Connectivity analyses were performed using a validated structural equation modeling method (SEM) (65) consistent with our previous studies (21, 31, 48, 56, 63–65). As cluster-to-cluster correlations may be insufficient to explain more complex coordination between regions (63, 64), this hypothesis-based data-driven method has been previously used to identify and characterize connectivity networks in the brain, brainstem and spinal cord (63, 64), identify coordinated networks during pain processing (32) and to explore changes in these networks during the expectation of pain (31). We have also previously demonstrated that these methods may be more effective for exploring differences in pain processing in chronic pain populations such as fibromyalgia compared to model-driven methods (66). SEM requires the use of a pre-defined model to describe possible connections between regions. Our model is illustrated in Figure 2 and includes information on anatomical directionality of connections.

SEM was carried out separately for the Expecting Pain and Experiencing Pain epochs, and each of the three groups (FM, PVD, HC). The method consists of fitting the BOLD time-series response in a “target” region to the BOLD responses in multiple “source” regions, in an effort to explain the BOLD response in the target as potentially arising from input signaling from the sources. The connectivity strength between regions was represented with a linear weighting factor (*β*, the relative contribution of each input to a region). For example, if region A receives input signaling from regions B and C, and the BOLD signal time-series responses in these regions are *S_A_*, *S_B_*, and *S_C_* respectively, then the BOLD signal variations in region A are represented as follows: *S_A_ = β_AB_ S_B + _β_AC_ S_C + _e_A_* where *e_A_* is the residual signal variation that is not explained by the fit (65). The model was divided into network components which consisted of multiple “source” regions (e.g., *S_B_*, S_C_) providing input to one “target region” (e.g., *S_A_*). The weighting factors (*β*) were calculated separately for each network component, and were calculated multiple times for each possible combination of clusters in each region, in order to determine which clusters provide the best fit. The amount of variance explained in each target region/cluster by the fit was expressed as an *R*^2^ value, and the corresponding significance was estimated by converting the *R* value to a Z-score using Fisher's Z-transform. The resulting Z-scores have a normal distribution from which the *p*-value was estimated. A cutoff corresponding to a family-wise error corrected significance threshold of *p* < 0.05 was used.

#### 2.5.4. Comparing and contrasting connectivity networks

To compare connectivity networks across the three groups and time periods, we employed analyses of covariance (ANCOVA) (66), using the participant group as a discreet independent variable (FM vs. PVD vs. HC) and participant normalized pain scores as a continuous independent variable. Normalized pain scores were calculated for each participant using the ratio of their pain ratings during each run to the temperature of the stimulus used to produce that pain rating (pain score = pain rating/temperature). A higher ratio (and therefore a higher normalized pain score) reflects higher pain sensitivity. This was done in order to standardize subjective measurements across participants and groups. Main effects of group, pain scores, and interaction effects were calculated for each of the time periods (Expecting and Experiencing Pain) and significance was inferred at a multiple-comparison-corrected significance threshold of *p* < 0.05. As the main effect of group included three levels (FM, PVD, and HC), any connections with a significant main effect of group were subsequently analyzed with Bonferroni-corrected post-hoc tests to verify which groups are significantly different from each other in each case.

## 3. Results

### 3.1. Participant characteristics

Questionnaire scores were compared among the FM, PVD, and healthy control groups with an ANOVA. Ages were significantly different among all three groups, *F*(2,59) = 9.88, *p* = 0.001, with the FM group being the oldest (mean age = 46.7 years), HC next (mean age = 34.4 years), and the PVD group being the youngest (mean age = 29.4 years). There was also a main effect of group for the pain catastrophizing scores, *F*(2,37) = 7.08, *p *= 0.002, with the HC group having lower pain catastrophizing scores than both the FM (*p *= 0.003) and PVD groups (*p* = 0.026). Pain catastrophizing was not significantly different between the two chronic pain groups. To examine any potential relationships between the different questionnaire scores, all questionnaires were also compared with a Pearson's correlation, and this was done separately for each of the three groups (Table 1).

**Table 1:** Comparison of questionnaire score relationships for the FM, PVD, and HC groups.

	FM	PVD	HC
Age vs. Normalized pain score	0.620*	0.069	−0.227
BDI vs. SF-MPQ-2	0.515*	−0.174	0.843**
BDI vs. PC	0.664*	0.705*	0.118
PC vs. Normalized pain score	0.409	0.661*	−0.087
BDI vs. STAI (state)	0.164	0.038	0.421*
BDI vs. STAI (trait)	0.332	0.040	0.564**

Numbers indicate Pearson's correlation coefficients and are indicated with one asterisk (*) if the relationship was significant at the *p* < 0.05 level, and two asterisks (**) if significant at the *p* < 0.001 level. Acronyms indicate depression scores (BDI), pain impact scores (SF-MPQ-2), pain catastrophizing scores (PC), and state and trait anxiety scores (STAI).

All relationships between all questionnaire scores, as well as age, were tested, but only the ones with a statistically significant *r* value in at least one group are displayed in Table 1. In the fibromyalgia group, age was positively correlated with the normalized pain score, and depression scores were positively correlated with both pain impact and pain catastrophizing scores. In the PVD group, depression scores were also positively correlated with pain catastrophizing scores, but pain catastrophizing was also positively correlated with the participants' age. In the healthy control group, depression scores were positively correlated with pain impact scores, and with both state and trait anxiety scores.

### 3.2. Comparison of fMRI results across groups

Connectivity networks in the brainstem and spinal cord examined by means of SEM analyses showed extensive interconnected networks in all three groups, for both the Expecting Pain and Experiencing Pain epochs. These results were subsequently used in ANCOVAs to compare connectivity strengths (*β*) across the three groups. ANCOVA analyses were performed separately for the Expecting Pain and Experiencing Pain timepoints. For each, an ANCOVA was performed with the participant group as a discreet independent variable (FM vs. PVD vs. HC) and normalized pain scores as a continuous independent variable. Several connections were identified with a significant main effect of group in both timepoints (Table 2).

**Table 2:** Group x normalized pain score ANCOVA results for connections which had a significant main effect of group.

	FM vs. PVD	FM vs. HC	PVD vs. HC
**Expecting Pain**			
Thalamus → PAG	*	*	*
LC → NGc	*	*	*
Hypothalamus → NTS	*	** **	** **
DRt → C6RD	** **	*	** **
PAG → NRM	*	*	** **
PBN → C6RD	*	*	** **
Hypothalamus → NRM	** **	** **	*
PAG → Hypothalamus	** **	*	** **
**Experiencing Pain**			
LC → Hypothalamus	*	** **	*
Hypothalamus → NGc	** **	** **	*
PBN → C6RD	*	*	*
LC → Thalamus	** **	** **	*
PBN → NGc	*	*	** **
Drt → C6RD	*	** **	*
PAG → NGc	*	*	*
PAG → Hypothalamus	*	** **	** **
NTS → Hypothalamus	** **	*	*
NRM → C6RD	** **	*	*
Hypothalamus → PAG	*	** **	** **
NTS → C6RD	*	*	** **
C6RD → NRM	** **	** **	*

Asterisks denote which post-hoc comparisons were statistically significant. For example, in the first connection listed (thalamus to PAG), *post hoc* tests indicate that connectivity strengths in this connection were significantly different between all three participant groups.

**Figure 3:**
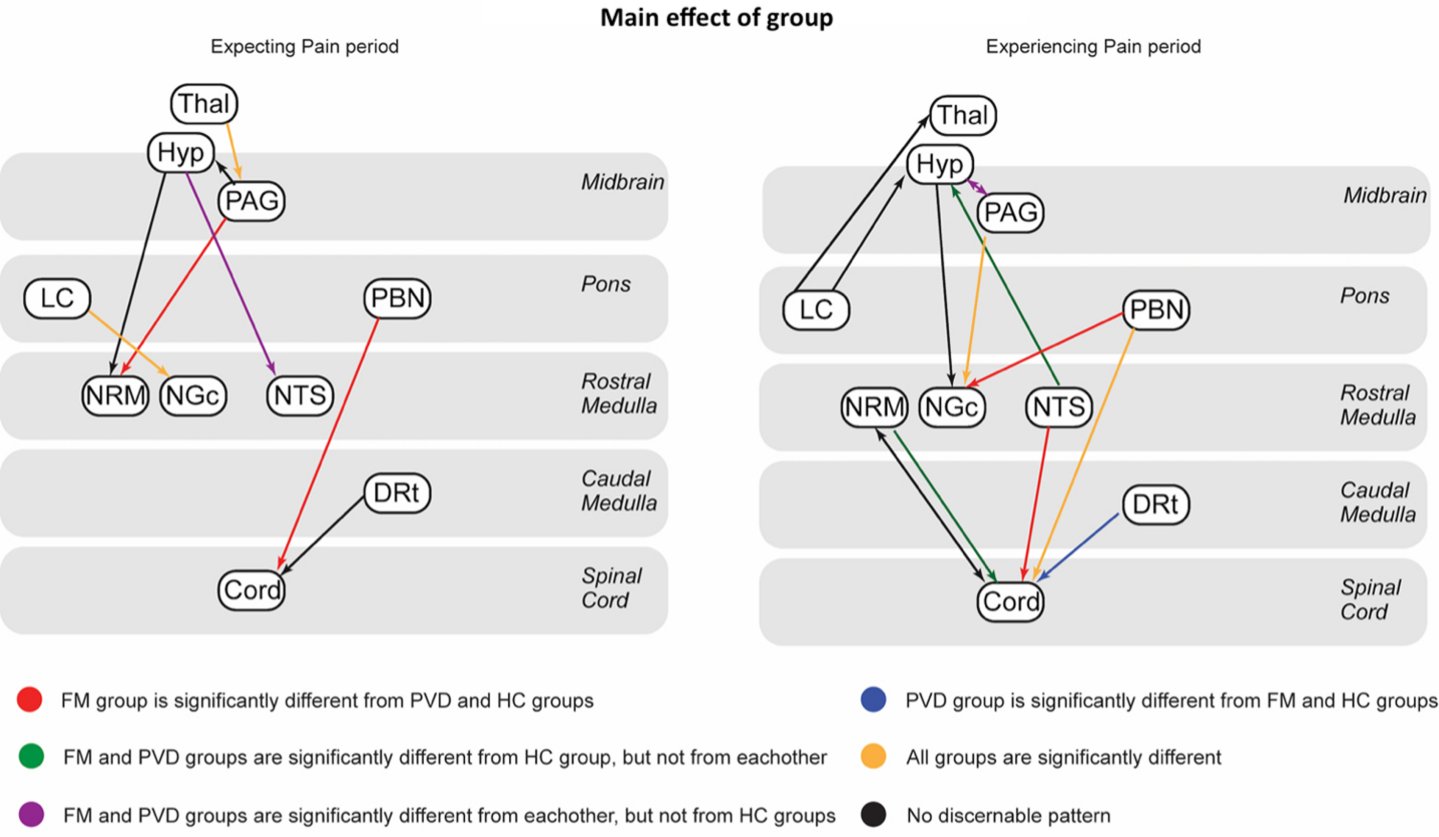
Summary of ANCOVA results showing connections with a significant main effect of group in the expecting pain (left) and experiencing pain (right) timepoint. Connections are color-coded based on which groups were significantly different from each other, as evaluated with post-hoc tests. Black lines indicate connections where no interpretable pattern was found. Details of all connections are available in Table 2 for reference.

A greater number of connections with a main effect of group identified in the Experiencing Pain timepoint compared to Expecting Pain, with several connections to and from the hypothalamus, and several connections with signaling between spinal cord and RVM regions. In contrast, connections with a main effect of group identified during the Expecting Pain time period consisted mainly of signaling to and from the hypothalamus, signaling from the thalamus to the PAG and from the PAG to the NRM, and signaling from areas such as the DRt and PBN to the spinal cord. These differences are depicted graphically in Figure 3. Connections which had a main effect of normalized pain score or an interaction effect in both timepoints are detailed in Table 3.

**Table 3:** Group x normalized pain score ANCOVA results highlighting connections with a significant main effect of normalized pain score (above) and a significant interaction effect (below).

Expecting Pain	Experiencing Pain
**Main effect of normalized pain score**	
PAG → NRM	LC → NRM
PAG → LC	PBN → NTS
C6RD → NGc	LC → NGc
PAG → DRt	PBN → NRM
LC → PBN	PAG → NGc
LC → C6RD	PAG → NRM
PBN → C6RD	NTS → C6RD
Hypothalamus → LC	C6RD → NGc
C6RD → NRM	PBN → C6RD
** **	LC → NRM
** **	PBN → NTS
** **	LC → NGc
**Interaction effect**	
Hypothalamus → NGc	PAG → NGc
PBN → NGc	LC → NRM
PAG → NGc	C6RD → NGc
C6RD → NGc	Hypothalamus → LC
PBN → NTS	Hypothalamus → NTS
LC → C6RD	PAG → Hypothalamus
LC → DRt	Hypothalamus → NGc
NRM → LC	C6RD → Thalamus
PAG → LC	PBN → NGc
PAG → Hypothalamus	NTS → Hypothalamus
Hypothalamus → LC	NTS → LC
NTS → PBN	PAG → NRM

Arrows indicate the direction of the connection (source → target). The first column shows the results of the analysis performed for the Expecting Pain period, while the second column shows the results for the Experiencing Pain period (where participants were feeling the noxious heat stimulus).

**Figure 4:**
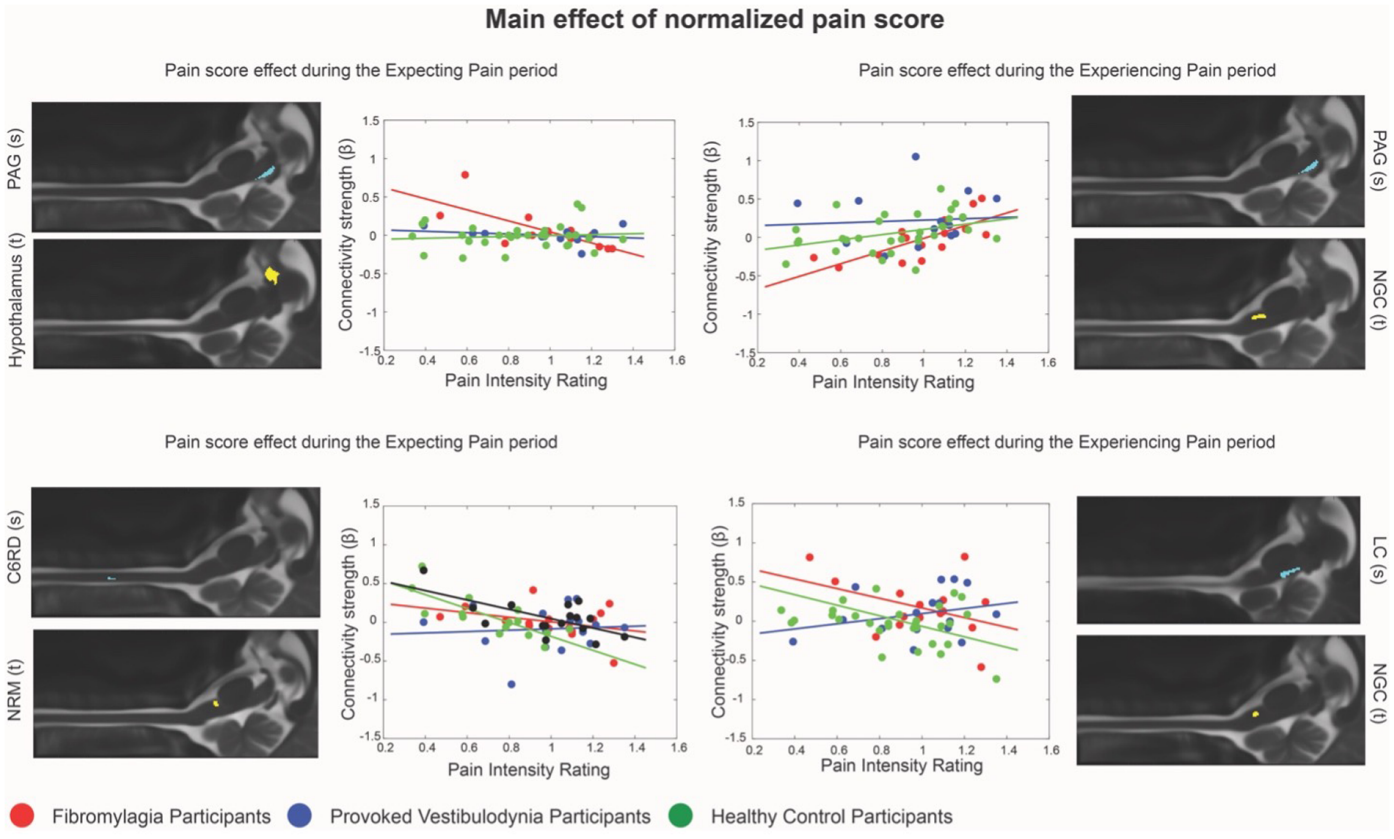
Examples of connections with a significant main effect of normalized pain score. Each panel presents an anatomical image indicating the source (above, blue, marked with s) and target region (below, yellow, marked with t) for each given connection. Each graph shows the relationship between the connectivity strength (*β*) and normalized pain score for each individual and group. Participants from the FM group are marked in red, the PVD group is marked in blue, and the HC group is marked in green, with a matching colour trendline for each group. Four connections are shown. Connections in the left column had a significant main effect of normalized pain score during the Expecting Pain period, while the right column is during the Experiencing Pain period. The upper panels feature connections where the trend in the FM group was different from the PVD and HC groups, while the lower panels show connections where the trend in the PVD group was different from the FM and HC groups.

Fewer connections were identified with a main effect of the normalized pain score in the Expecting Pain compared to the Experiencing Pain time period, while there were a similar number of connections with interaction effects during both times. Connections with main effects of pain scores were relatively similar between timepoints, with more signaling from the hypothalamus to the brainstem in the Expecting Pain period, and more LC and PBN connections to other brainstem areas while Experiencing Pain. Interaction effects were also generally consistent between the two epochs, with more connections between the PBN and other brainstem areas having interaction effects while Expecting Pain, and more connections between the hypothalamus and brainstem areas having interaction effects while Experiencing pain. Detailed examples are given in Figure 4, showing the connectivity strength variation with normalized pain scores for two connections during the Expecting Pain, and Experiencing Pain period.

## 4. Discussion

The results of the present study build on previous efforts to characterize chronic overlapping pain conditions (67) by showing important commonalities and differences in connectivity among participants with fibromyalgia and provoked vestibulodynia. SEM analyses revealed extensive connectivity networks in the Expecting Pain and Experiencing Pain time periods for all three groups (FM, PVD, and HC). These results revealed several connections with significant group differences in both time periods (Figure 3) and showed elements of network connectivity that are consistent between the two chronic pain populations. In the Expecting Pain condition, there were no significant differences in connectivity strengths in the connections DRt → cord, hypothalamus → NRM, and PAG → hypothalamus between the FM and PVD groups. However, there were significant connectivity differences between the HC group and either the PVD or FM group in these connections (Table 2). While connectivity between the PAG and RVM is associated with descending pain modulation (68), integration of signaling to and from the hypothalamus in this pathway may indicate that these networks are linked to autonomic homeostatic regulation as described by Craig (62). During the Experiencing Pain condition, consistent connectivity between the FM and PVD groups included LC → thalamus, NTS → hypothalamus, and NRM → cord. Signaling between the NRM and spinal cord is generally associated with descending pain modulation (17), while connectivity between regions such as the thalamus, hypothalamus, and NTS may also be attributed to integration of autonomic regulation with pain regulation (62). Additionally, involvement of the LC and NTS in these results may also infer effects of arousal or salience of the stimulus (32, 69–71). These indicate that consistent effects of FM and PVD on pain modulation may be attributed in part to integration of autonomic regulation networks with descending pain modulation pathways.

Interestingly, signaling between the DRt and spinal cord has been previously described as a feedback loop related to pain modulation, where DRt activity contributes to regulation of the responsiveness of spinal cord neurons to noxious stimulation (72). Both FM and PVD pain have been speculated to involve central sensitization (9–21), and several studies have demonstrated that individuals with these chronic pain conditions show increased temporal summation of pain compared to healthy controls (12, 18, 20, 21). The current results support those findings and show that activity in this DRt-spinal cord feedback loop is consistent between FM and PVD, lending support to the idea that both conditions may be linked to changes in the regulation of spinal cord sensitivity to pain. It must be noted, however, that this effect was only seen during the Expecting Pain period, and not during noxious stimulation. We have previously demonstrated that pain modulation includes a continuous component that is present even before a painful stimulus is applied (32). This may indicate that continuous pain modulation during the expectation of pain is similar between FM and PVD and includes modulation of spinal cord sensitivity when anticipating predictable pain.

While PVD are categorized as chronic pain conditions primarily affecting women, FM is a generalized chronic pain condition associated with widespread musculoskeletal pain (2–4), and PVD is further characterized by provoked pain in the vulvar region (5, 6). The results of the present study provide evidence of similarly altered pain processing in FM and PVD compared to HC, despite differences in pain location and clinical presentation between these conditions. In this context, it is especially interesting to consider connections where there was a significant main effect of group with no differences in connectivity between FM and PVD groups, but where both groups were significantly different from the HC group (Figure 3, green). It is likely that these effects are related to the patients' chronic pain, because the results of both conditions are significantly different from those of healthy participants. This only occurred during stimulation, in the connections NTS → hypothalamus and NRM → cord. Based on our previous studies and the known functions of these regions (21, 31–33, 62, 64, 68), it is possible that these connectivity changes indicate altered integration of autonomic and pain regulation systems in these conditions. Both FM and PVD have been previously shown to involve autonomic dysfunction (22, 23, 25, 26), and this link between the two conditions can now be supported with data from brainstem/spinal cord fMRI. While there were only two connections where this effect was identified, these provide the first evidence of changes in pain regulation that may be associated to broader effects of chronic pain across FM and PVD, rather than one specific pain condition.

As individuals with FM and PVD have different pain histories and clinical presentations, differences in descending signaling from several limbic regions, which are integrated with pain processing networks at the level of the brainstem and spinal cord, can be expected. A large portion of the connections where a significant main effect of group was identified showed differences in connectivity strength between FM and PVD groups (Figure 4). A subset of connections where all three groups significantly differed included thalamus → PAG and LC → NGC in the Expecting Pain time period, as well as PAG → NGC and PBN → cord in the Experiencing Pain period. These effects again suggest a potential integration of descending pain modulation systems (17) with other networks including regions involved in autonomic regulation and arousal (32, 62, 69–71). There were also similar trends identified within the interaction effects, indicating that network connectivity in the brainstem and spinal cord varies with individual normalized pain scores in both chronic pain groups, but that this effect involves different connections in FM compared to PVD. Interestingly, FM and PVD participants had similar normalized pain scores and both group averages were significantly higher than the HC pain scores. Many of the connections identified in these effects involve the descending pain modulation network (68) as well as parts of the interoception and homeostatic regulation networks described by Craig (62). These differences therefore likely relate to differing mechanisms underlying FM and PVD pain.

A large proportion of the connections with trends that differed in the FM group compared to PVD and HC included regions involved with autonomic homeostatic regulation such as the hypothalamus, NTS, and PBN (62). Although both FM and PVD have been described to include some autonomic dysfunction (22, 23, 25, 26), the results in Table 2 and Figure 3 show more connections related to autonomic regulation where connectivity is linked to changes in pain sensitivity in FM than in PVD. Additionally, the influence of autonomic regulation within pain modulation networks may affect normalized pain scores differently in individuals with FM compared to PVD or HC. These ideas are supported by previous research which has linked changes in pain sensitivity to autonomic regulation changes in FM (23, 25, 26). In contrast, connections where trends differed in the PVD group compared to the FM and HC groups included several number of connections with the LC, which may be involved with effects of arousal or salience on pain modulation (32, 69–71).

Although both FM and PVD participants had similar pain catastrophizing scores (which were significantly higher than in the HC groups), pain catastrophizing was significantly correlated with normalized pain scores only in the PVD group. The painful stimulus used in this study elicits acute pain, which individuals with PVD may experience more often than FM participants who usually experience widespread diffuse pain (2–4). However, stimulation was applied to the hand, where participants with FM more often report pain (2–4) compared to PVD participants whose pain is localized primarily to the vulvar region (5, 6). While we cannot determine if this stimulus was consistently more salient for FM or PVD participants and why, the questionnaire scores indicate that attitudes towards pain are linked to individual normalized pain scores in PVD participants but not in FM. This may, in turn, be linked to connectivity differences between PVD and FM in brainstem networks integrating arousal and autonomic regulation effects with pain modulation, where connectivity strength varies with normalized pain scores in different subsets of the networks depending on the chronic pain group.

## 5. Limitations

SEM analyses require a model of possible connections including directionality information as a basis for the analyses. As a result, there may be some regions relevant to the effects we discuss that were not included in the initial model (Figure 2). When performing such analyses we must strike a balance between including enough relevant regions to be able to observe any effects relevant to our hypothesis questions while limiting the amount of regions and information included in order to reduce the complexity of the model and the number of comparisons made. Our current model is based on several anatomical atlases, imaging resources, and prior literature on pain processing in the human brain and brainstem and has been employed in numerous peer-reviewed research studies. However, it is possible that other relevant effects exist that are not discussed or illustrated here.

## 6. Conclusions

This is the first study to compare FM and PVD groups with fMRI data from the spinal cord and brainstem which has shed light on differences in descending pain modulation in these conditions. We have demonstrated similarities in pain processing at the level of the brainstem and spinal cord while expecting and experiencing a painful stimulus, indicating common elements for how pain processing is altered in chronic pain conditions. We have also shown differences in pain processing between these two conditions, which have important implications for our understanding of both FM and PVD. Our results indicate that differences in arousal and salience may be uniquely linked to pain processing in PVD, and that connectivity in these brainstem/spinal cord networks varies with normalized pain scores in more regions involved autonomic regulation in FM than in PVD. This evidence is an important step toward understanding how pain processing is altered in chronic pain, as well as uncovering unique characteristics of FM and PVD pain processing respectively.

## Data Availability

The raw data supporting the conclusions of this article will be made available by the authors, without undue reservation.
